# Planning a One Health Case Study to Evaluate Methicillin Resistant *Staphylococcus aureus* and Its Economic Burden in Portugal

**DOI:** 10.3389/fmicb.2018.02964

**Published:** 2018-12-07

**Authors:** Gilberto Igrejas, Susana Correia, Vanessa Silva, Michel Hébraud, Manuela Caniça, Carmen Torres, Catarina Gomes, Fernanda Nogueira, Patrícia Poeta

**Affiliations:** ^1^Department of Genetics and Biotechnology, University of Trás-os-Montes and Alto Douro, Vila Real, Portugal; ^2^Functional Genomics and Proteomics Unit, University of Trás-os-Montes and Alto Douro, Vila Real, Portugal; ^3^LAQV-REQUIMTE, Faculty of Science and Technology, University Nova of Lisbon, Lisbon, Portugal; ^4^Veterinary Science Department, University of Trás-os-Montes and Alto Douro, Vila Real, Portugal; ^5^Université Clermont Auvergne, Institut National de la Recherche Agronomique, UMR0454 MEDiS, Centre Auvergne-Rhône-Alpes, Saint-Genès-Champanelle, France; ^6^Institut National de la Recherche Agronomique, Plate-Forme d’Exploration du Métabolisme Composante Protéomique, UR0370 QuaPA, Centre Auvergne-Rhône-Alpes, Saint-Genès-Champanelle, France; ^7^National Reference Laboratory of Antibiotic Resistances and Healthcare Associated Infections, Department of Infectious Diseases, National Institute of Health Dr. Ricardo Jorge, Lisbon, Portugal; ^8^Área de Bioquímica y Biología Molecular, Universidad de La Rioja, Logroño, Spain; ^9^Área de Microbiología Molecular, Centro de Investigación Biomédica de La Rioja, Logroño, Spain; ^10^Centro de Administração e Políticas Públicas, Instituto Superior de Ciências Sociais e Políticas, Universidade de Lisboa, Lisbon, Portugal

**Keywords:** antimicrobial resistance, surveillance, MRSA, One Health, omics

## Abstract

Methicillin-resistant *Staphylococcus aureus* (MRSA) is one of the most important multidrug-resistant nosocomial pathogens worldwide with infections leading to high rates of morbidity and mortality, a significant burden to human and veterinary clinical practices. The ability of *S. aureus* colonies to form biofilms on biotic and abiotic surfaces contributes further to its high antimicrobial resistance (AMR) rates and persistence in both host and non-host environments, adding a major ecological dimension to the problem. While there is a lot of information on MRSA prevalence in humans, data about MRSA in animal populations is scarce, incomplete and dispersed. This project is an attempt to evaluate the current epidemiological status of MRSA in Portugal by making a single case study from a One Health perspective. We aim to determine the prevalence of MRSA in anthropogenic sources liable to contaminate different animal habitats. The results obtained will be compiled with existing data on antibiotic resistant staphylococci from Portugal in a user-friendly database, to generate a geographically detailed epidemiological output for surveillance of AMR in MRSA. To achieve this, we will first characterize AMR and genetic lineages of MRSA circulating in northern Portugal in hospital wastewaters, farms near hospitals, farm animals that contact with humans, and wild animals. This will indicate the extent of the AMR problem in the context of local and regional human-animal-environment interactions. MRSA strains will then be tested for their ability to form biofilms. The proteomes of the strains will be compared to better elucidate their AMR mechanisms. Proteomics data will be integrated with the genomic and transcriptomic data obtained. The vast amount of information expected from this omics approach will improve our understanding of AMR in MRSA biofilms, and help us identify new vaccine candidates and biomarkers for early diagnosis and innovative therapeutic strategies to tackle MRSA biofilm-associated infections and potentially other AMR superbugs.

## Introduction

*Staphylococcus aureus* is a Gram-positive facultative anaerobe frequently present in the natural human microbiota of the nose and skin that can cause a range of illnesses from minor skin infections and food poisoning to life-threatening diseases such as pneumonia, toxic shock syndrome and sepsis (Sousa et al., 2017). The first methicillin-resistant *S. aureus* (MRSA) was reported only a year after the introduction of methicillin for *S. aureus* treatment (Jevons, 1961). MRSA is resistant to almost all beta-lactams and frequently carries other major classes of antimicrobial resistance (AMR).

Most AMR research has been focused on bacteria growing in planktonic cultures and antimicrobials were originally developed to target individual bacterial cells. However, it is clear that bacteria preferentially develop as complex communities called biofilms (Seneviratne et al., 2012; Penesyan et al., 2015). Recent advances in proteomics techniques have enabled a more in-depth analysis of the possible mechanisms responsible for biofilm AMR and the identification of new anti-biofilm targets (Seneviratne et al., 2012; Azeredo et al., 2017). The use of prefractionation techniques to extract subproteomes significantly enhanced protein identification and coverage of the biofilm proteome (Seneviratne et al., 2012). Also, new shotgun proteomics workflows based on high-resolution tandem mass spectrometry (MS/MS) directly coupled to high performance liquid chromatography (LC) require less protein than conventional two-dimensional gel electrophoresis (2-DE) approaches, allowing a more exhaustive analysis of proteomes or subproteomes and the performance of label-free semi-quantitative comparisons (Azeredo et al., 2017).

Staphylococci have for many decades been recognized as the most frequent cause of biofilm-associated infections (Cihalova et al., 2015; McCarthy et al., 2015). Since the 1990s the epidemiological profile of MRSA has been changing significantly. Its emergence is no longer exclusive to hospitals, as the prevalence of community-acquired infections is increasing (European Centre for Disease Prevention and Control [ECDC], 2017a,b). In fact, several cases of people having had no contact with hospital environments have been diagnosed with MRSA despite having no risk factors for contracting an infection by these organisms (Sousa et al., 2017). In recent years, new genetic lineages of MRSA have been found associated with companion (Leonard and Markey, 2008; Coelho et al., 2011), livestock and food-producing animals, and in various foods (Lee, 2003). However, there is little information on how MRSA spreads and data about the strains recovered from environmental sources, animals and human communities is far from comprehensive. Convergences between habitats can lead to frequent contact between wild animals, other animals and humans, potentially increasing risks to human and animal health. For example, human sources of AMR determinants could contaminate surrounding areas used as food sources for wild animals. More MRSA strains are expected to emerge in the future. The implementation of measures to control zoonotic pathogens and limit the global emergence of resistance traits is required. Integration of human and veterinary systems alone is insufficient as it does not address many structural and environmental issues critical to health.

Biofilm-associated infections are a significant socio-economic burden and have emerged as a major public health concern (Sun et al., 2013; Penesyan et al., 2015). Nearly 80% of all human infections are biofilm-related and one of their most critical features is their considerably higher resistance to environmental stresses, antimicrobials, disinfectants and host immune defenses (Seneviratne et al., 2012; Sun et al., 2013). Despite major advances in biofilm research, knowledge on biofilm formation, propagation and resistance is still very limited and this poor understanding has hampered the development of antimicrobial drugs that specifically target biofilms (Penesyan et al., 2015; Venkatesan et al., 2015).

Antimicrobial resistance acquisition and dissemination rates are outpacing the drug development pipeline (Harbarth et al., 2015; O’Neill, 2016). AMR has the potential to affect anyone of any age in any country (World Health Organization, 2014). If not adequately addressed, AMR could cause 10 million deaths and cost 100 trillion dollars by 2050 (O’Neill, 2014, 2016; European Commission [EC], 2017). Patients with drug-resistant infections or diseases tend to consume more resources and are sick for longer periods, increasing the risk of severe outcomes even if they manage to overcome their main health issue. In addition, the families and entourage of the ill person also end up suffering on personal, practical and economic levels (Ellen et al., 2017). The continuous quantification of the economic burden of these diseases on the individual and on society in general will show the direct consequences of AMR on health system budgets, and other costs that might be associated with losses incurred by different stakeholders (e.g., patients, carers, and governments) (Angelis et al., 2015).

When making such estimates the perspective being taken when considering such scenarios needs to be well defined (Naylor et al., 2018). The payer/provider perspective juxtaposes the patient’s perspective, which concerns itself with morbidity, mortality and the clinical outcomes, and the payer’s perspective, which focuses on healthcare costs attributable to medical insurance and tax payers (Naylor et al., 2018). The healthcare provider’s perspective also needs to be taken into account to estimate the burden on some providers of healthcare like hospitals and primary care practices. Finally, the economic or societal perspective generally includes the potential impact on the labor force through decreases in productivity, but also the burden on carers and patient out-of-pocket expenses (Naylor et al., 2018). There may be secondary effects of AMR if certain healthcare procedures involving antimicrobial usage are avoided. In a systematic literature review, Naylor and Colleagues (2018) found 187 studies estimated the impact on patient health, 75 studies estimated the payer/provider impact and 11 studies estimated the economic burden. Overall 64% of the studies reviewed were single-center studies. The great majority of studies estimating patient or provider/payer impact used regression analyses. AMR was found to have a significant impact in 48% of the studies that estimated mortality burden. Excess healthcare system costs ranged from non-significant to $1 billion per year, whereas economic burden varied from $21,832 per case to over $3 trillion in GDP loss. Median quality scores (interquartile range) for patient, payer/provider and economic burden studies were 0.67 (0.56–0.67), 0.56 (0.46–0.67), and 0.53 (0.44–0.60), respectively. AMR has therefore become a cause of international concern not only due to the actual and future impact it may have on the population’s health, but also on the costs to healthcare systems and gross domestic product (GDP), mainly by the decrease in treatment options.

This project will aim first to provide a better understanding of MRSA prevalence, burden and dissemination in the One Health context of human-animal-environment interactions and then to investigate through proteomics the AMR mechanisms occurring in MRSA biofilms. By characterizing AMR and genetic lineages of MRSA circulating in anthropogenic sources in the North of Portugal, this project will provide epidemiological surveillance data, compiled and easily accessible to the scientific community, public health officials, and the general public. The further proteomic profiling of MRSA biofilms will increase our knowledge of biofilm-specific AMR mechanisms and identify potential vaccine candidates and biomarkers for early rapid diagnosis and new therapeutic strategies.

## Materials and Methods

### Samples

Samples from hospital effluents, and nearby habitats linked to animal farms and wild animal territories will be collected in the Portuguese north province of Trás-os-Montes and Alto Douro annually. Specifically:

(a) Hospital samples will be obtained from the four public hospitals responsible for the public health of the citizens of this province – [Hospital Centre of Trás-os-Montes and Alto Douro (CHTMAD)] – these are located in the cities of Lamego, Peso da Régua, Chaves, and Vila Real. Ethical approval and support has been granted by CHTMAD.
(b) Farm samples will be obtained from soils and farmers, and from animals and their handlers from all the 31 municipalities of the province. The representativeness of the sample of farms will be calculated after obtaining the data referring to the type of farms existing by municipality (e.g., cows, pigs, birds, etc.), and subsequent randomization of the animal samples of each farm.
(c) Wild animal samples will be collected by groups of hunters during the wild rabbit and wild boar hunts and by the Wildlife Recovery Center (Centro de Recuperação de Animais Selvagens, CRAS) at the University of Trás-os-Montes and Alto Douro veterinary hospital.

This data will allow us to map, characterize, and monitor AMR and genetic lineages of MRSA annually, by its presence or absence in the area. It will also indicate the extent of the problem of local and regional human-animal-environment interactions in Trás-os-Montes e Alto Douro (One Health and Eco Health Concepts).

Furthermore, questionnaires regarding the use of antibiotics by the participating entities will be sent to them for annual update – (e.g., hospitals: how many antibiotics have been prescribed by this entity in the last year?; farms: how many of your animals have been administrated antibiotics? How many times has that occurred in the last year?).

### MRSA Detection

*Staphylococcus aureus* and MRSA will be recovered on mannitol salt agar and oxacillin resistance screening agar base (ORSAB), respectively. Presumptive *S. aureus* and MRSA colonies will be identified based on their morphology and re-isolated. Their identity will be confirmed by genotyping using molecular methods and VITEK technology, via PCR amplification of the *nuc* and *mecA* genes. Phenotypic antimicrobial susceptibility will be tested with the EUCAST disk diffusion and broth microdilution methods and the presence of corresponding resistance genes will be investigated by PCR and sequencing. The clonal relationship of isolates will be assessed by pulsed-field gel electrophoresis, *spa*-typing, *agr*-typing and multilocus-sequence-typing (MLST).

### Data Collection

All data will be compiled and added to a new web-based application developed so that georeferenced AMR data can be consulted and visualized by medical professionals, the scientific community and others that require it. Existing data on antibiotic resistant staphylococci in Portugal will also be compiled and included in the database. Similar interfaces exist such as the ECDC’s Surveillance Atlas of Infectious Diseases and the CDC’s Antibiotic Resistance Patient Safety Atlas that allow users to openly interact and manipulate AMR data to customize a variety of maps and tables. However, finer granularity is intended with the possibility to retrieve and filter data at the level of sample collection and isolation details, AMR phenotypic and genotypic profiles, genetic lineages, biofilm-forming ability, among others. Additionally, this information should be traceable to available proteomic, genomic and transcriptomic data of the individual MRSA strains.

Importantly, as AMR prediction and surveillance spans many scientific realms (public health, research, agriculture, drug discovery, etc.), ease-of-use translational tools and data sharing are increasingly needed, requiring a collective dedication to standardization. Although several global surveillance programs exist that monitor AMR (McArthur and Tsang, 2017), genotypic data is not found in their datasets and accessible databases that combine genotypic and phenotypic AMR data for pathogens in environmental, agricultural, and clinical settings are still not available. Hence, the generation of these informatics resources are of high priority considering their value for epidemiology, antimicrobial stewardship, and drug discovery (McArthur and Tsang, 2017).

### Analysis of the Outcomes

**FIGURE 1:**
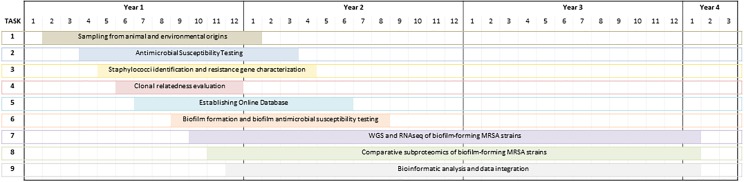
Timeline of case study based on the comparative assessment of antimicrobial resistance of MRSA isolates from Portugal.

All isolated MRSA strains will be tested for their ability to form biofilms. The antimicrobial susceptibility of any biofilm-forming strains will be re-assessed. The proteomes of a number of biofilm-forming MRSA strains will then be characterized. Different subproteomes of MRSA biofilms will be analyzed and compared using both electrophoretic and direct mass spectrophotometric approaches (2-DE-LC-MS/MS and shotgun LC-MS/MS) to identify differentially expressed proteins induced by antimicrobials. The strains selected for proteomic analyses will also be characterized at the genomic and transcriptomic level by whole genome sequencing and RNA sequencing. All omics data will be analyzed and integrated using bioinformatics tools. Several institutions including universities and laboratories will cooperate in this data integration task and all the collaborating international research groups will provide support for data interpretation (Figure 1). Biosafety standards will be respected at all stages of the work.

## Preliminary and Expected Results

### One Health Focus on Livestock-Associated MRSA in Portugal and Europe

In the past decade, our research group has been surveying AMR in bacteria from a great diversity of environments, collecting over 4,000 samples from more than 75 different sources (humans, wastewaters, food-producing animals, pets, and wild animals), amounting to over 5,000 bacterial isolates. High levels of AMR to critically important drug classes and high rates of clinically relevant multi-resistant strains in non-synanthropic animal species have been found (Marinho et al., 2016). Portugal is one of the countries with highest rates of MRSA and about 44% of the Portuguese hospital *S. aureus* isolates are methicillin-resistant, the second highest rate in Europe (European Centre for Disease Prevention and Control [ECDC], 2017a,b). Our recently published research reveals that MRSA are common in the Portuguese animal communities (Coelho et al., 2011; Marinho et al., 2016) and that the environment and wild animals can be a reservoir or a vehicle of transport for MRSA (Sousa et al., 2017). Currently our research group is one of the few in Portugal that studies antibiotic resistance in wild animals (Oliveira et al., 2010; Clemente et al., 2015; Dias et al., 2015; Jones-Dias et al., 2016; Serrano et al., 2017). For example, we reported the first MRSA isolate of the CC398 (*spa*-type t899) lineage from a wild animal in this country, recovered from a wild boar (*Sus scrofa*). The isolate is *agr*-type I and carries a multi-antibiotic-resistance phenotype, including against beta-lactams (*mec*A gene), tetracycline and ciprofloxacin (Sousa et al., 2017).

We do not know how AMR flows through the environment. This proposal is the next step to investigate the flow of AMR in MRSA and to establish a publicly available, user-friendly database that compiles and integrates the new and existing data on MRSA in Portugal. To illustrate the approach, we can take the spread of the livestock-associated MRSA (LA-MRSA) ST398 as an example. MRSA are indeed becoming frequent in veterinary clinics, in farms, and in livestock animals. In recent years, this particular MRSA clone associated with food production has spread in Europe and is emerging worldwide. Since its discovery, there has been a steady flow of reports of LA-MRSA ST398 among livestock, especially pigs, in numerous European countries (Loeffler et al., 2009). People exposed to livestock are at greater risk of being colonized, and subsequently, infected with LA-MRSA ST398, especially if they are working on farms with a high prevalence. The occupational risk for people exposed to livestock, and those in direct contact with them, has been repeatedly shown. There is not enough data to compare studies in Portugal alone, but if studies from other countries are considered, we can say that LA-MRSA infections may occur outside and independently of hospitals (Pomba et al., 2010). LA-MRSA CC398 is able to cause the same kind of infections that human-adapted MRSA (HA-MRSA) causes in humans. Comparative genome analysis has shown that LA-MRSA has evolved from HA-MRSA, and the jump from humans to livestock has been clearly associated with several genetic changes (Price et al., 2012). We will further analyze the proteome and the transcriptome associated to this strain and compare it to the online data on CC398 strains to confirm whether this is an LA-MRSA or a genetically distinguishable strain with zoonotic potential originating from wild animals. Whatever the result, the evolution and re-adaptation of these bacteria to various animal or human populations pose a potential health risk requiring close surveillance.

Beyond surveillance studies, our research group has investigated AMR mechanisms by characterizing a range of resistant strains of interest through proteomic approaches in MRSA (Monteiro et al., 2012, 2015) and other bacterial species (Pinto et al., 2010; Radhouani et al., 2010, 2012; Correia et al., 2014, 2016; Goncalves et al., 2014; Ramos et al., 2015, 2016; Monteiro et al., 2016). However, these proteomic studies, and most AMR research in general, have focused on bacteria growing in planktonic cultures and hence overlooked biofilm-specific AMR mechanisms. These are known to be distinct from the well-characterized intrinsic mechanisms that occur at the cellular level, operating additively to the latter, in a transient and reversible manner, resulting in up to 1000-fold higher resistance levels (Sun et al., 2013; Penesyan et al., 2015; Azeredo et al., 2017). Hence, biofilm-specific mechanisms need to be considered when developing new strategies to combat infectious diseases (Sun et al., 2013; Penesyan et al., 2015).

**FIGURE 2:**
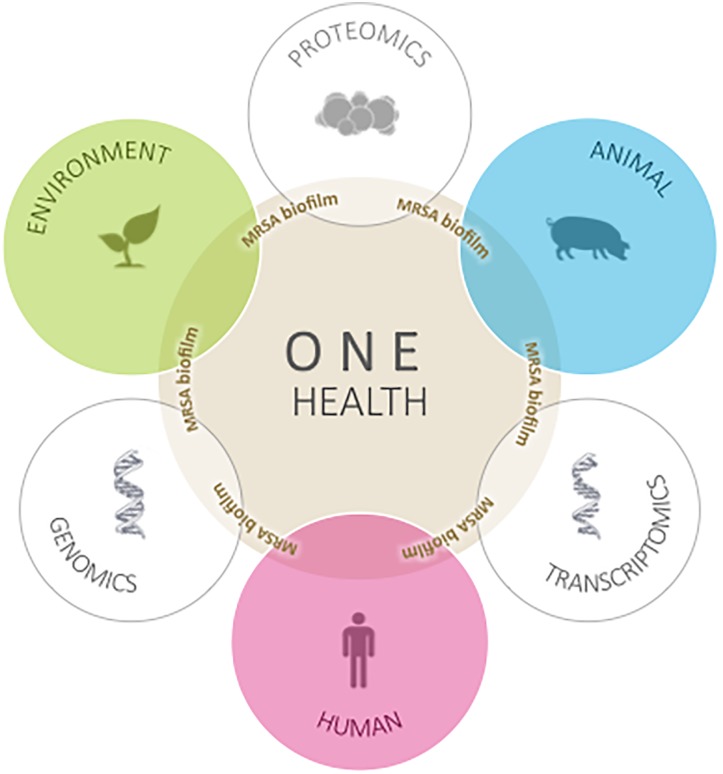
Graphical abstract of case study based on the comparative assessment of antimicrobial resistance of MRSA isolates from Portugal.

The objective is to promote collaboration between several public entities as well as different stakeholders from industry and media. We aim to produce information by studying AMR in bacteria from wild animals with zoonotic potential. The potential impacts are both internal, by generating more precise knowledge and collaboration, and external, by improving animal, human, and environmental health in the long term (Figure 2). All the MRSA data generated by studying isolates from wild animals will be disseminated according to the principles of One Health information sharing.

### Scientific Tasks and Challenges

All *S. aureus* strains should be isolated and MRSA strains identified. Phenotypic and genotypic AMR profiles of all strains should be determined, and molecular typing of strains should be completed. The database should be online and functional. All data on methicillin-sensitive *S. aureus* and MRSA isolates characterized in this project, together with existing data from isolates from Portugal, should be compiled in the database. Testing for biofilm formation and antimicrobial susceptibility should be completed for all MRSA isolates. Strains for further omics approaches should be chosen.

When considering the MRSA issue, we automatically think about outbreaks in the clinical setting. The truth is that this worrisome organism is everywhere, in human clinical isolates, in healthy people who work in clinical or care facilities, in livestock animals and their handlers, in food production and slaughter lines, in wastewater, garbage, and as recently shown, in wild animals. This situation is clearly not new and not wholly unexpected, but in Portugal this environmental MRSA flow is problematic. Our research team has been trying to draw attention to this issue by centering our investigations on the veterinary and environmental aspects. As well as the knowledge and expertise we have accumulated within our team, factors that contributed positively to this work were the collaborations between laboratories and associations, and the individual scholarships from the Portuguese Foundation for Science and Technology (FCT) that made it possible for our students to carry out field work and have access to specialized laboratories. Many factors were challenging at the outset like collecting the samples, often in bad weather conditions. However, with the cooperation of six faculties, stakeholders from industry and media, and Portuguese governmental initiatives we are continuing our surveillance of AMR bacteria recovered from wild animal populations.

### Strategy for One Health Knowledge Sharing

Two strategic axes have been established to reduce the risk of AMR caused by the use of antibiotics in animals. The aim of the public health protection axis is to reduce the impact of administered veterinary antibiotics on AMR spread. The therapeutic preservation axis is designed to promote the sustainability and efficacy of antimicrobial use. Our plan reflects EU policies because the opinions of a wide range of stakeholders have been taken into account with input from academia and industry, and from practitioners like veterinarians, pharmacists and farmers. One Health learning is expected to involve individual researchers and institutions by the creation of long-term supportive interdisciplinary infrastructures and professional networks.

Effective communication is essential to underpin such a wide-ranging approach. Regular scientific meetings for consortium updates will complement international congresses to disseminate and discuss findings. Engagement will extend into the community through lectures to high school and university students, and take advantage of social and traditional media outlets. Partners in the countryside will be targeted by providing educational workshops for hunters and cooperating with the League for Nature Protection. Professional guidelines and good practice will be observed, disseminated, promoted and reinforced for all practitioners (distributors, veterinarians, and farmers). For example, active participation in the National Action Plan for Antibiotics Use Reduction in Animals will help to trace and validate veterinary prescription and requisition and to harmonize the register of all medicines administered at farms. With adequate support and training of all professions dealing with animal health and animal production, better selection and use of antibiotics will be promoted, and innovations and alternatives can be explored.

The promotion of investigation, innovation and technological exchanges to incentivize the development of alternative means of treatment, whenever possible and a reinforced monitoring, audits and controls are very important actions to fight against AMR. Research outcomes will be of high quality as we will find out which AMR bacterial variants are associated with each focus of infection and each animal species in a particular habitat. A galvanized network of specialists should more able to prompt the authorities to take action to better regulate antibiotic prescription in hospitals and care facilities (for humans and animals) and on farms, and to take control over how antimicrobials are disposed of, especially when there is a risk of polluting the environment.

Antimicrobial resistance is estimated to cause 25,000 deaths annually and cost over €1.5 billion in healthcare expenses and productivity losses in Europe alone (O’Neill, 2014; European Commission [EC], 2017). In general, higher resistance frequencies are reported by countries in eastern and southern Europe (European Centre for Disease Prevention and Control [ECDC], 2017b). Given the severity of the consequences, MRSA is now a public health priority in Europe and is also one of the highest-priority pathogens in the WHO global priority list to guide research, discovery and development of new antibiotics. The high incidence of MRSA adds to the overall clinical and economic burden in hospitals, causing prolonged hospital stays and higher mortality, mainly due to delayed initiation of appropriate therapy and less effective alternative treatment regimens (O’Neill, 2016; European Centre for Disease Prevention and Control [ECDC], 2017a,b). Given this, there is an impetus to understand the Portuguese situation in more depth, and precision. To facilitate uptake of results and meta-analysis we will base our research on the recommendations of Naylor et al. (2018) by clearly defining data collection and use wherever possible from representative samples of the population studied. Potential confounding factors and biases will be carefully considered when choosing the methodology. All steps of data collection and processing will be clearly recorded. Wider impacts on healthcare systems and economics will be estimated where possible with explanation and justification of any models chosen (Table 1).

**Table 1:** Proposed One Health activities, aims and monitoring to implement and integrate knowledge to evaluate the current methicillin resistant *Staphylococcus aureus* situation and estimate its economic burden at the formulation stage of the policy cycle.

One Health initiative specific aims regarding five main activities	Monitoring^1^ (transversal activity)	One Health initiative main aim
Thinking	• Stipulate the dimensions that need coverage, and balance different areas of knowledge and multiple perspectives. • Reflect upon the initiative-to-environment match. • Reflect upon the best integrated health approach. • Consider all the system features and targets and, sustainability and socio-ecological-economic impacts. • Think and decide upon relevant performance indicators for each One Health Initiative main activity.		

Planning	• Establish common aims that will lead to stakeholder and actor engagement while monitoring, self-assessing and updating each plan when needed.	During all the initiative phases the following are of extreme relevance to be under monitoring and assessment for the need for change, development and, innovation:	

Working	• Stimulate the inclusion and collaboration of each stakeholder of the initiative, broadening its impact while balancing its transdisciplinary (cultural, social, and economic) – nature.	• Research Problem and Design • Team structures • Social and leadership structure • Social and leadership skills • Competence/Skills • Resource allocation • Focus and innovation	Set guidelines that allow for Policy Formulation. (This is possible through the results obtained during the five activities. These will allow evidence which will point out solutions to the AMR problem, and recommendations on how to improve its situation.)

Sharing	• Stimulate systematic general information/awareness sharing, through data and information sharing and development of methods and stimulation of results sharing. These activities intend to develop institutional memory and resilience ability.

Learning	• Create a general and direct multilevel (individual level, team level and organizational level) learning environment supportive of adaptive and generative learning.

*^*1*^Implies monitoring and assessing the processes and results implied in each suggested dimension implied in each initiative activity. This is intended to stimulate the initiatives reflexivity and adaptiveness ability. Adapted from Hitziger et al. (2018)*.

## Discussion

### One Health Initiative to Address AMR in Portugal

The One Health approach is the European Commission strategy to tackle AMR, as it recognizes that the health of people, animals and the environment are inextricably linked. This project intends to answer several One Health evaluation questions. (i) How can the spread of AMR be avoided in both human and veterinary medicine? (ii) How can we define the role of wildlife in AMR gene flow? (iii) What steps should we advocate to disseminate our future results? And (iv) How should this issue be addressed in terms of public health?

By involving different universities and stakeholders from industry this project has a One Health attitude from the outset. Different scientific work packages will address the following topics: isolation and identification of strains; genomic and genotypic studies; demographic and socioeconomic characterization; sequencing studies; phenotypic studies; proteomic and transcriptomic analysis; results verification and homologation.

This project will consolidate knowhow in the isolation and identification of MRSA strains from different ecosystems in Portugal. Several collaborations will be maintained and developed between different research groups through this and other projects. This will allow the creation of a bacterial collection with hundreds of strains comprehensively analyzed with genomics and proteomics tools. Knowledge and expertise in using these tools to characterize AMR bacteria will be consolidated, particularly in genotyping techniques by enterobacterial repetitive intergenic consensus PCR and MLST. Purified bacteriocins will be characterized biochemically by MALDI-TOF MS, N-terminal amino acid sequencing by Edman degradation, and sequencing by MALDI TOF/TOF MS. The use of these techniques and the associated equipment will allow us to establish standard protocols for proteomics.

The results of this investigation may add to our knowledge on the occurrence of MRSA strains and the genetic lineages circulating in our surroundings. A more precise local estimate of AMR due to the MRSA burden can inform policy and shape the initiatives to monitor, prevent, treat and limit the spread of resistant infections.

The potential impacts of this case study will lead to better knowledge and collaboration in our interdisciplinary consortium and extended network as well as improvements in animal, human and environmental health.

This project builds on previous efforts of European Commission programs and other programs worldwide and aims to answer priority questions in research and innovation for infectious diseases. First, AMR, genetic lineages and biofilm-forming ability of MRSA strains circulating in anthropogenic sources will be characterized, adding to the emerging picture of the extent of the AMR problem in the context of human-animal-environment interactions. Data will be made available in a free, user-friendly online platform, providing a geoepidemiological output. Further proteomic profiling of MRSA biofilms, integrated with high-throughput genomics and transcriptomics, will provide a large amount of data that will extend the currently limited knowledge on biofilm-specific AMR mechanisms. If this is successful, new molecular candidates for vaccines or biomarkers will be identified that could be developed for early rapid diagnosis and innovative therapeutic strategies to tackle biofilm-associated infections in MRSA and other superbugs with high burden impact. Such an investment in research and innovation taking into consideration the multi-layered burdens of MRSA will improve prevention and treatment and will help us to remain active and vigilant, to develop new, safer and more effective medical treatments, to maintain health and to ensure the viability of health systems. Hopefully this will stimulate more concerted action to reduce the prevalence of MRSA and AMR in Portugal and further afield.

## Author Contributions

GI, SC, VS, CG, FN, and PP wrote the manuscript. MH, MC, and CT helped design the case study. GI and PP conceived the review. All authors reviewed and contributed to the manuscript.

## Conflict of Interest Statement

The authors declare that the research was conducted in the absence of any commercial or financial relationships that could be construed as a potential conflict of interest.

## References

[B1] Angelis A. Tordrup D. Kanavos P. (2015). Socio-economic burden of rare diseases: a systematic review of cost of illness evidence. *Health Policy* 119 964–979. 10.1016/j.healthpol.2014.12.016 25661982

[B2] Azeredo J. Azevedo N. F. Briandet R. Cerca N. Coenye T. Costa A. R. (2017). Critical review on biofilm methods. *Crit. Rev. Microbiol.* 43 313–351. 10.1080/1040841X.2016.1208146 27868469

[B3] Cihalova K. Chudobova D. Michalek P. Moulick A. Guran R. Kopel P. (2015). *Staphylococcus aureus* and MRSA growth and biofilm formation after treatment with antibiotics and SeNPs. *Int. J. Mol. Sci.* 16 24656–24672. 10.3390/ijms161024656 26501270 PMC4632770

[B4] Clemente L. Manageiro V. Jones-Dias D. Correia I. Themudo P. Albuquerque T. (2015). Antimicrobial susceptibility and oxymino-beta-lactam resistance mechanisms in *Salmonella enterica* and *Escherichia coli* isolates from different animal sources. *Res. Microbiol.* 166 574–583. 10.1016/j.resmic.2015.05.007 26054292

[B5] Coelho C. Torres C. Radhouani H. Pinto L. Lozano C. Gomez-Sanz E. (2011). Molecular detection and characterization of methicillin-resistant *Staphylococcus aureus* (MRSA) isolates from dogs in Portugal. *Microb. Drug Resist.* 17 333–337. 10.1089/mdr.2010.0080 21254810

[B6] Correia S. Hebraud M. Chafsey I. Chambon C. Viala D. Torres C. (2016). Impacts of experimentally induced and clinically acquired quinolone resistance on the membrane and intracellular subproteomes of *Salmonella* Typhimurium DT104B. *J. Proteomics* 145 46–59. 10.1016/j.jprot.2016.04.001 27063838

[B7] Correia S. Nunes-Miranda J. D. Pinto L. Santos H. M. de Toro M. Saenz Y. (2014). Complete proteome of a quinolone-resistant *Salmonella* Typhimurium phage type DT104B clinical strain. *Int. J. Mol. Sci.* 15 14191–14219. 10.3390/ijms150814191 25196519 PMC4159846

[B8] Dias D. Torres R. T. Kronvall G. Fonseca C. Mendo S. Caetano T. (2015). Assessment of antibiotic resistance of *Escherichia coli* isolates and screening of *Salmonella* spp. in wild ungulates from Portugal. *Res. Microbiol.* 166 584–593. 10.1016/j.resmic.2015.03.006 25869224

[B9] [EC] (2017). *Antimicrobial Resistance: Commission Launches Public Consultation on New Action Plan*, ed. HaF S. (Brussels: European Commission).

[B10] Ellen M. E. Hughes F. Shach R. Shamian J. (2017). How nurses can contribute to combating antimicrobial resistance in practice, research and global policy. *Int. J. Nurs. Stud.* 71 A1–A3. 10.1016/j.ijnurstu.2017.02.023 28318533

[B11] European Centre for Disease Prevention and Control [ECDC] (2017a). *Antimicrobial Resistance Surveillance in Europe 2015. Annual Report of the European Antimicrobial Resistance Surveillance Network (EARS-Net)*. Stockholm: European Centre for Disease Prevention and Control.

[B12] European Centre for Disease Prevention and Control [ECDC] (2017b). *Antimicrobial Resistance Surveillance in Europe 2016. Annual Report of the European Antimicrobial Resistance Surveillance Network (EARS-Net)*. Stockholm: European Centre for Disease Prevention and Control.

[B13] Goncalves A. Poeta P. Monteiro R. Marinho C. Silva N. Guerra A. (2014). Comparative proteomics of an extended spectrum beta-lactamase producing *Escherichia coli* strain from the Iberian wolf. *J. Proteomics* 104 80–93. 10.1016/j.jprot.2014.02.033 24631823

[B14] Harbarth S. Balkhy H. H. Goossens H. Jarlier V. Kluytmans J. Laxminarayan R. (2015). Antimicrobial resistance: one world, one fight! Antimicrobial resistance and infection control. *Antimicrob. Resist. Infect. Control* 4:49 10.1186/s13756-015-0091-2

[B15] Hitziger M. Esposito R. Canali M. Aragrande M. Häsler B. Rüegg S. R. (2018). Knowledge integration in One Health policy formulation, implementation and evaluation. *Bull. World Health Organ.* 96 211–218. 10.2471/BLT.17.202705 29531420 PMC5840631

[B16] Jevons M. P. (1961). “Celbenin” - resistant Staphylococci. *Br. Med. J.* 1 124–125.

[B17] Jones-Dias D. Manageiro V. Graca R. Sampaio D. A. Albuquerque T. Themudo P. (2016). QnrS1- and Aac(6’)-Ib-cr-Producing *Escherichia coli* among isolates from animals of different sources: susceptibility and genomic characterization. *Front. Microbiol.* 7:671 10.3389/fmicb.2016.00671 PMC4876607 27242699

[B18] Lee J. H. (2003). Methicillin (Oxacillin)-resistant *Staphylococcus aureus* strains isolated from major food animals and their potential transmission to humans. *Appl. Environ. Microbiol.* 69 6489–6494. 14602604 10.1128/AEM.69.11.6489-6494.2003 PMC262320

[B19] Leonard F. C. Markey B. K. (2008). Meticillin-resistant *Staphylococcus aureus* in animals: a review. *Vet. J.* 175 27–36. 10.1016/j.tvjl.2006.11.008 17215151

[B20] Loeffler A. Kearns A. M. Ellington M. J. Smith L. J. Unt V. E. Lindsay J. A. (2009). First isolation of MRSA ST398 from UK animals: a new challenge for infection control teams? *J. Hosp. Infect.* 72 269–271. 10.1016/j.jhin.2009.04.002 19481297

[B21] Marinho C. M. Santos T. Goncalves A. Poeta P. Igrejas G. A. (2016). Decade-long commitment to antimicrobial resistance surveillance in Portugal. *Front. Microbiol.* 7:1650. 10.3389/fmicb.2016.01650 27843438 PMC5086874

[B22] McArthur A. G. Tsang K. K. (2017). Antimicrobial resistance surveillance in the genomic age. *Ann. N. Y. Acad. Sci.* 1388 78–91. 10.1111/nyas.13289 27875856

[B23] McCarthy H. Rudkin J. K. Black N. S. Gallagher L. O’Neill E. O’Gara J. P. (2015). Methicillin resistance and the biofilm phenotype in *Staphylococcus aureus*. *Front. Cell. Infect. Microbiol.* 5:1 10.3389/fcimb.2015.00001 PMC4309206 25674541

[B24] Monteiro R. Hebraud M. Chafsey I. Chambon C. Viala D. Torres C. (2015). Surfaceome and exoproteome of a clinical sequence type 398 methicillin resistant *Staphylococcus aureus* strain. *Biochem. Biophys. Rep.* 3 7–13. 10.1016/j.bbrep.2015.07.004 29124163 PMC5668672

[B25] Monteiro R. Hebraud M. Chafsey I. Poeta P. Igrejas G. (2016). How different is the proteome of the extended spectrum beta-lactamase producing *Escherichia coli* strains from seagulls of the Berlengas natural reserve of Portugal? *J. Proteomics* 145 167–176. 10.1016/j.jprot.2016.04.032 27118263

[B26] Monteiro R. Vitorino R. Domingues P. Radhouani H. Carvalho C. Poeta P. (2012). Proteome of a methicillin-resistant *Staphylococcus aureus* clinical strain of sequence type ST398. *J. Proteomics* 75 2892–2915. 10.1016/j.jprot.2011.12.036 22245554

[B27] Naylor N. R. Atun R. Zhu N. Kulasabanathan K. Silva S. Chatterjee A. (2018). Estimating the burden of antimicrobial resistance: a systematic literature review. *Antimicrob. Resist. Infect. Control* 7:58. 10.1186/s13756-018-0336-y 29713465 PMC5918775

[B28] Oliveira M. Pedroso N. M. Sales-Luis T. Santos-Reis M. Tavares L. Vilela C. L. (2010). Antimicrobial-resistant *Salmonella* isolated from Eurasian otters (Lutra lutra Linnaeus, 1758) in Portugal. *J. Wildl. Dis.* 46 1257–1261. 10.7589/0090-3558-46.4.1257 20966276

[B29] O’Neill J. (2014). *Antimicrobial Resistance: Tackling a Crisis for the Health and Wealth of Nations*. Available at: http://www.jpiamr.eu/wp-content/uploads/2014/12/AMR-Review-Paper-Tackling-a-crisis-for-the-health-and-wealth-of-nations_1-2.pdf

[B30] O’Neill J. (2016). *Tackling Drug-Resistant Infections Globally: Final Report and Recommendations*. San José, CA: Inter-American Institute for Cooperation on Agriculture.

[B31] Penesyan A. Gillings M. Paulsen I. T. (2015). Antibiotic discovery: combatting bacterial resistance in cells and in biofilm communities. *Molecules* 20 5286–5298. 10.3390/molecules20045286 25812150 PMC6272253

[B32] Pinto L. Poeta P. Vieira S. Caleja C. Radhouani H. Carvalho C. (2010). Genomic and proteomic evaluation of antibiotic resistance in *Salmonella* strains. *J. Proteomics* 73 1535–1541. 10.1016/j.jprot.2010.03.009 20346428

[B33] Pomba C. Baptista F. M. Couto N. Loucao F. Hasman H. (2010). Methicillin-resistant *Staphylococcus aureus* CC398 isolates with indistinguishable ApaI restriction patterns in colonized and infected pigs and humans. *J. Antimicrob. Chemother.* 65 2479–2481. 10.1093/jac/dkq330 20829201

[B34] Price L. B. Stegger M. Hasman H. Aziz M. Larsen J. Andersen P. S. (2012). *Staphylococcus aureus* CC398: host adaptation and emergence of methicillin resistance in livestock. *mBio* 3:e00305-11. 10.1128/mBio.00305-11 22354957 PMC3280451

[B35] Radhouani H. Pinto L. Poeta P. Igrejas G. (2012). After genomics, what proteomics tools could help us understand the antimicrobial resistance of *Escherichia coli*? *J. Proteomics* 75 2773–2789. 10.1016/j.jprot.2011.12.035 22245553

[B36] Radhouani H. Poeta P. Pinto L. Miranda J. Coelho C. Carvalho C. (2010). Proteomic characterization of *van*A-containing *Enterococcus* recovered from Seagulls at the Berlengas Natural Reserve, W Portugal. *Proteome Sci.* 8:48. 10.1186/1477-5956-8-48 20858227 PMC2954869

[B37] Ramos S. Chafsey I. Silva N. Hebraud M. Santos H. Capelo-Martinez J. L. (2015). Effect of vancomycin on the proteome of the multiresistant *Enterococcus faecium* SU18 strain. *J. Proteomics* 113 378–387. 10.1016/j.jprot.2014.10.012 25449832

[B38] Ramos S. Silva N. Hebraud M. Santos H. M. Nunes-Miranda J. D. Pinto L. (2016). Proteomics for drug resistance on the food chain? multidrug-resistant *Escherichia coli* proteomes from slaughtered pigs. *OMICS* 20 362–374. 10.1089/omi.2016.0044 27310477

[B39] Seneviratne C. J. Wang Y. Jin L. Wong S. S. Herath T. D. Samaranayake L. P. (2012). Unraveling the resistance of microbial biofilms: has proteomics been helpful? *Proteomics* 12 651–665. 10.1002/pmic.201100356 22246638

[B40] Serrano I. Oliveira M. Santos J. P. Bilocq F. Leitao A. Tavares L. (2017). Antimicrobial resistance and genomic rep-PCR fingerprints of *Pseudomonas aeruginosa* strains from animals on the background of the global population structure. *BMC Vet. Res.* 13:58. 10.1186/s12917-017-0977-8 28222788 PMC5319083

[B41] Sousa M. Silva N. Manageiro V. Ramos S. Coelho A. Goncalves D. (2017). First report on MRSA CC398 recovered from wild boars in the north of Portugal. Are we facing a problem? *Sci. Total Environ.* 59 26–31. 10.1016/j.scitotenv.2017.04.054 28412568

[B42] Sun F. Qu F. Ling Y. Mao P. Xia P. Chen H. (2013). Biofilm-associated infections: antibiotic resistance and novel therapeutic strategies. *Future Microbiol.* 8 877–886. 10.2217/fmb.13.58 23841634

[B43] Venkatesan N. Perumal G. Doble M. (2015). Bacterial resistance in biofilm-associated bacteria. *Future Microbiol.* 10 1743–1750. 10.2217/fmb.15.69 26517598

[B44] World Health Organization (2014). *Antimicrobial Resistance: Global Report on Surveillance.* Geneva: World Health Organization.

